# The Interplay between Multimorbidity, Physical Work Demands and Work Ability: Cross-Sectional Study among 12,879 Senior Workers

**DOI:** 10.3390/ijerph19095023

**Published:** 2022-04-20

**Authors:** Karina Glies Vincents Seeberg, Sebastian Venge Skovlund, Rúni Bláfoss, Kristina Thomassen, Lasse Malchow-Møller, Emil Sundstrup, Lars Louis Andersen

**Affiliations:** 1 National Research Centre for the Working Environment, 2100 Copenhagen, Denmark; svs@nfa.dk (S.V.S.); rub@nfa.dk (R.B.); krt@nfa.dk (K.T.); xlam@nfa.dk (L.M.-M.); esu@nfa.dk (E.S.); lla@nfa.dk (L.L.A.); 2 Research Unit for Muscle Physiology and Biomechanics, Department of Sports Science and Clinical Biomechanics, University of Southern Denmark, 5230 Odense, Denmark; 3 Department of Health Science and Technology, Aalborg University, 9220 Aalborg, Denmark

**Keywords:** multimorbidity, musculoskeletal diseases, mental disorder, somatic disease, chronic disease, workers, work ability, workplace

## Abstract

Background: Aging increases the risk of chronic diseases, which can challenge the ability to work and thereby push senior workers out of the labour market. This study investigates the association between non-communicable diseases (NCDs) and work ability among workers ≥50 years (senior workers) with physically demanding and sedentary work, respectively. Methods: In the SeniorWorkingLife study, 12,879 senior workers replied to a questionnaire survey on work and health in 2018. Associations between the type and number of NCD and work ability (scale 0–10) were modelled using a general linear model adjusting for potential confounders and combined with model-assisted weights from national registers. Results: A higher number of NCD (multimorbidity) was progressively associated with a lower work ability (trend test, *p*-value < 0.001). Physical work influenced the association between the number of NCDs and work ability. For specific diseases, mental disorders, including burn-out syndrome (least square mean difference (LSMD): −1.46, 95% CI: −1.61 to −1.32) and stress ( LSMD: −1.18, 95% CI: −1.29 to −1.07), demonstrated a stronger association with a lower work ability compared with somatic diseases, such as back diseases (LSMD: −0.72, 95% CI: −0.80 to −0.64). Conclusions: Multimorbidity was progressively associated with a lower work ability in senior workers, especially among those with physical work.

## 1. Introduction

The increased life expectancy in many European countries has resulted in a demographic shift towards a larger elderly population. As a consequence, most countries have implemented political reforms to gradually increase the state pension ages. In Denmark, the retirement age will rise from the current 66.5 years in 2021 to 69 years by 2035 [1]. Thereby, more senior workers are expected to stay in the labour market as they age, which calls for an increased focus on sustainable employment throughout the working life [1,2,3].

Ageing is associated with the increased prevalence and incidence of non-communicable diseases (NCDs) [4,5]. There are several ways to define non-communicable disease (NCD) [6]. The World Health Organization (WHO) defines NCD as diseases that do not pass from person to person, are typically of long duration, progress slowly, and are often a combination of genetic, physiological, environmental, and behavioural factors [7]. NCD accounted for 71% of all deaths globally in 2021 [7], with the most common in Europe being cardiovascular diseases, neuropsychiatric conditions, cancer (malignant neoplasms), and respiratory diseases [4].

Having NCD is an important risk factor for leaving the labour market both temporarily in terms of absence due to sickness or unemployment [8] or permanently through early retirement and disability pension [8,9]. Multimorbidity is the co-occurrence of two or more NCDs. Multimorbidity is particularly prevalent in the older population and is an even stronger predictor for both partial and full-time retirement, including disability pension, increased sick leave, and lower quality of life compared with suffering from a single NCD [10,11,12,13,14].

NCDs are furthermore associated with reduced work ability [13], which in turn is associated with productivity loss, long-term sickness absence, and early retirement [4,8,15,16,17,18]. Physical work demands may be another factor influencing work ability among workers with NCD [19,20]. Work ability is defined as the balance between individual resources and the demands of work [21,22,23,24]. Additionally, poor work ability combined with one or more NCDs was found to be associated with a higher risk of long-term sickness absence [25].

Physically demanding work may feel more strenuous for workers with certain NCD, such as back diseases and in particular, for those with multimorbidity, i.e., symptoms can make it difficult to cope with the physical work demands. Some studies indicate that physical work negatively influences work ability among workers with musculoskeletal pain [19,20]. Specifically, neck pain was associated with a lower work ability among older workers with physically demanding work [20], and high physical demands at work were associated with a lower work ability among workers with musculoskeletal leg pain [19].

Identifying “vulnerable” groups of workers, i.e., those with low work ability, is important to preventing early involuntary exits from the labour market. However, the prevention should not only be targeted at the individual level, but it is highly important to prevent at a societal and company level as companies benefit from keeping healthy workers, which can positively impact productivity and contribute to sustainable employment [26].

To strengthen the opportunity for a long and healthy working life among workers in different types of jobs, more knowledge is needed regarding the association between work ability and type and number of NCDs (mental, physical, and multimorbidity) as well as the interplay between physical work demands and NCD with work ability. To the best of our knowledge, this study is the first to investigate the influence of physical work demands on the association between NCD and work ability and to investigate the association between different types and numbers of NCD and work ability among workers with different types of jobs.

Specifically, in the present study, we sought to investigate (1) associations between multimorbidity and work ability among workers ≥50 years (senior workers) and (2) whether work demands influence the association between the number of NCD and work ability. We hypothesised that having multiple NCDs would be associated with lower work ability compared to only having one NCD, and that workers with a combination of physically demanding work and multiple NCDs would demonstrate lower work ability than sedentary workers with multiple NCDs. As a secondary objective, we also explored the association between different types of chronic disease (mental and somatic) and work ability.

## 2. Materials and Methods

### 2.1. Study Design and Settings

This cross-sectional study employed data from the first wave of the SeniorWorkingLife study, which is registered as a cohort study in ClinicalTrials.gov (identifier; NCT03634410). The SeniorWorkingLife study is a questionnaire survey about work environments and health among senior workers (≥50 years) in Denmark [27]. The first wave was sent out in July 2018 and collected in October 2018 [27]. This study follows the ‘Strengthening the Reporting of Observational Studies in Epidemiology’ (STROBE) guidelines [28], and the specific questions used in this study are specified below.

### 2.2. Participants and Eligibility Criteria

Statistics Denmark drew a probability sample of 30,000 Danes aged 50 years or older, i.e., senior workers (18,000 employed, 7000 unemployed, 3000 on voluntary early retirement, and 2000 on disability pension) [27]. The participants received an invitation to participate via their digital mailbox (‘E-boks’) with a web link to the questionnaire survey [27]. In this study, we included currently employed workers who were 50 years or older in Denmark. The response percentage for completing all questions in the survey was 56%. 

We also included participants who did not complete the entire questionnaire, and therefore the exact number of participants in each analysis varies. In total, we included 12,879 participants in the study. As the questionnaire included more questions than used in the present article, the individual respondents could easily have missing responses for some of the questions. Furthermore, to account for non-response and to make the estimates representative, we used model-assisted weights.

### 2.3. Ethical Considerations

Statistics Denmark de-identified the individual responses and stored the data on a secure server. The researchers performed all the analyses through remote access. Danish law permits the scientific usage of questionnaires and registers data without applying for approval by ethical and scientific committees and without collecting informed consent.

### 2.4. Explanatory Variables

#### 2.4.1. Non-Communicable Diseases

The WHOs definition was used to define NCD in this study and NCDs were identified by the following question: *Have you within the past year received treatment or medication for one or more of the following diseases/disorders? Anxiety, Asthma, ischemia, apoplexy, depression, diabetes all types, eczema, hypertension, cataract, chronic obstructive pulmonary disease, rheumatoid arthritis, cancer, peptic ulcers, migraine/headache, hearing impairment, osteoporosis, whiplash, back diseases, osteoarthritis, stress, burn-out syndrome, or other long-term sickness/illness.*

The response options for each disease were “*Yes*” and “*No*” [27].

#### 2.4.2. Physical Work Demands

The following question assessed physical work demands: *How would you generally describe your physical activity level in your current job?* The response options were: *(1) Mostly sedentary work that is not physically demanding. (2) Mostly standing and walking work that otherwise is not physically demanding. (3) Standing or walking work with some lifting and carrying tasks. (4) Heavy or fast work that is physically demanding* [29,30,31]. For the subsequent analyses, the last three response options were pooled and categorised as ‘physical work’, whereas the first response option was categorized as ‘sedentary work’.

### 2.5. Outcome Variables

#### Work Ability

Work ability was assessed by the following validated single-item question from the Work Ability Index (WAI): *Please rate your current work ability on a scale of 0–10, where 0 is unable to work and 10 is lifetime best/highest work ability* [32,33].

### 2.6. Confounders

We controlled the analyses for the following potential confounders: sex (“Male” and “Female”, categorical), age (years, continuous), highest completed education ((1) Primary school or unknown, High school (2) Short-term higher education, Medium-term higher education, and (3) Long-term higher education (categorical)), physical activity at work (see description above, categorical), physical activity during leisure (mostly sedentary, light exercise at least 4 hours per week, sports or heavy physical activity at least 4 hours per week, or training and competing regularly and several times per week, categorical), smoking status (“Yes, every day”, “yes, but not every day”, “ex-smoker”, or “No, never”, categorical), and body mass index (BMI (kg/m^2^, continuous)). Psychosocial work factors (influence at work and recognition from manager (continuous)) were assessed on a scale from 0 to 100 with questions originating from the Copenhagen Psychosocial Questionnaire (COPSOQ) [34].

### 2.7. Statistical Analyses

All analyses were performed in the SAS statistical software package (SAS v.9.4, SAS Institute, Cary, NC, USA). We used model-assisted weights based on national registers to make the estimates representative of ≥50 year workers in Denmark. General linear models were used to analyse the association between chronic disease as the independent variable and work ability as the dependent variable, which was numerical i.e., 0–10. First, the difference in work ability between those with and without disease was modelled separately for each chronic disease and controlled for age and gender. 

Next, we conducted two models for the number of NCDs. The first model adjusted for sex, age, education, and physical demands at work. The second model additionally adjusted for physical activity during leisure, smoking, BMI, and psychosocial work factors. The interaction between number of NCDs and physical work demands was included in both models. The results are presented as the least square means and least square mean differences of work ability and 95% confidence intervals (95% CI). An alpha level of *p* < 0.05 was considered as a statistically significant difference.

## 3. Results

### 3.1. Baseline Characteristics

Table 1 presents the demographic, lifestyle, diseases, and work-related characteristics of the included 12,879 employed senior workers (≥50 years). The mean age was 57 years, 53% were male, and 53% characterised their work as “mostly physically demanding work”, while 47% characterized their work as “mostly sedentary work”. The mean number of NCDs was 1.2 diseases. The workers rated their work ability to be 7.95 out of 10 on average. Almost a third (31%) of the sample reported two or more NCDs, i.e., multimorbidity.

### 3.2. Specific NCD and Work Ability

For the entire group of workers, having a chronic disease was associated with a lower work ability. More specifically, lower work ability was reported among workers with mental disorders, such as burnout syndrome (least square mean difference (LSMD): −1.46, 95% CI: −1.61 to −1.32), stress (least square mean difference: −1.18, 95% CI: −1.29 to −1.07), and anxiety (least square mean difference: −1.15, 95% CI: −1.31 to −0.99) as well as somatic diseases, such as back diseases (least square mean difference: −0.72 95% CI: −0.80 to −0.64) and ischemic heart disease (−0.69, 95% CI: −0.86 to −0.52) (Table 2).

### 3.3. Physical Work Demands, Number of NCD(s), and Work Ability

A statistically significant interaction existed in both models between the number of diseases and physical work demands for work ability. Work demands influenced the association between the number of NCDs and work ability (interaction: model 1: *p* = 0.0057, model 2: *p* = 0.0029). Therefore, the results of Table 3 are stratified by work demands. In model 2, for no NCD, the work ability results were 8.44 and 8.19 (mean diff. 0.25) in those with sedentary and physical work, respectively, and these values decreased to 7.20 and 6.59 (mean diff.: 0.60) for those with four NCDs.

## 4. Discussion

The main finding of the present study is that work demands influenced the association between the number of NCDs and work ability. Thus, we observed lower work ability among workers with a combination of high physical work demands and multiple NCDs. The study confirmed that many types of NCDs (i.e., both mental and somatic) are associated with lower work ability.

### 4.1. NCD and Work Ability

Our data confirm the previously reported high prevalence of NCD among seniors [4,5]. Of the included senior workers, 29% had at least one NCD, whereas 32% suffered from multiple co-occurring NCDs of either the mental or somatic kind. Mental NCD, especially anxiety, stress, and burnout syndrome, showed the strongest negative associations with work ability compared to other mental disorders and somatic diseases. In opposition, workers with asthma, hypertension, cataract, and eczema reported the highest work ability among the workers with NCD. It can be speculated that proper and effective medication and/or other treatment or disease characteristics could explain why workers with certain NCD retain higher work ability despite their NCD; however, these are hypotheses that are currently not supported by the data but that will have to be verified in the future.

Generally, the present study reports lower work ability among workers with mental disorders compared to those with somatic diseases. These findings are consistent with other studies reporting stronger negative associations between mental disorders and the ability to perform their work [13,35].

### 4.2. Multimorbidity and Work Ability

Among both physical and sedentary workers, a higher number of NCDs was associated with lower work ability compared to having only one disease. Furthermore, we observed a difference in the work ability between job groups when comparing the same numbers of diseases. The lowest work ability was observed in workers with physical work and four or more diseases. Hence, and consistent with our hypothesis, suffering from multiple NCDs instead of one disease may substantially challenge the ability to work. This is problematic from a European societal perspective and warrants action granted that 28–34% per cent of the European population is estimated to suffer from multimorbidity [36]. The most frequent combination of multimorbidity in 12 selective countries was osteoarthritis and cardiovascular and/or metabolic conditions [37].

It is likely that some combinations of co-occurring diseases may be (1) particularly complex to treat and handle and thus (2) particularly detrimental for the work ability compared to other combinations. Unfortunately, our data do not allow drawing inferences on the existence of particularly challenging combinations of NCD in terms of the impact on work ability, although this would provide a more sophisticated picture of complex constellations of diseases particularly worth focusing on. While our study can function as a foundation for mapping different associations between CND and work ability in different types of workers, future studies are required to verify our hypothesis of the causal inferences.

The duration of an NCD diagnosis and the severity of symptoms may also affect the impact on work ability. First, many patients’ first medical consultation may occur when their symptoms are at the most intense stage, and therefore their work ability may be highly inhibited [38]. Secondly, living with a chronic disease for many years results in increased knowledge of and action competencies to live and cope with the condition, e.g., medication, lifestyle, and work accommodations. For instance, a life-long diabetic may be better adapted and medicated compared with a newly diagnosed diabetic [38].

### 4.3. Physical Work Demands and Work Ability

Our study elaborates on previous findings demonstrating lower work ability among workers with multiple NCDs. Whereas both mental and somatic health diseases were associated with a lower work ability, mental health problems, in particular, were negatively associated with work ability [39,40]. The novel part of the present study is the significant interaction between physical work demands and NCD with work ability. In line with these findings, other studies have found that a combination of high physical work demands and pain significantly increases the odds of experiencing work limitations due to pain among senior workers [29,30,41]. Therefore, it is important to prioritize the physical work environment in physically demanding jobs as NCD affects these workers more in relation to their work ability compared to sedentary workers. One possible reason is that sedentary workers have better opportunities to adapt to their work in relation to their diseases [42]. 

For example, a sedentary worker with a back disease can adjust the working posture throughout the workday to regulate the pain intensity in order to maintain a high work ability, and the physical workload is too low to negatively affect the work ability to any large extent. This is more difficult for a worker with physically demanding job tasks, e.g. lifting tasks. However, future studies are required to verify this hypothesis.

The interaction between physical work demands and NCD with work ability may be explained by an imbalance between work capacity (low capacity due to diseases) and work demands (high due to physical strain). Adjusting work tasks to the worker’s physical or/and mental capacity could increase the work ability of the worker and help workers with multimorbidity continue working despite their disease. Another initiative to increase work ability is to increase physical capacity. Strength training, in particular, increases work ability among workers with physical jobs [43,44]. 

A systematic review investigated the effect of individual-focused interventions, including health assessment, advice on behaviour change, exercise programs, and developing active coping skills, compared to current practices, and the results indicated that none of the included studies of overall moderate quality reported a significant effect on work ability. It was proposed that this could be due to compliance issues [45]. Thus, conflicting evidence exists, and we require more prospective studies to clarify the conclusions.

The present results have important practical implications, i.e., companies should be aware that workers with chronic diseases might require adjustment of their work demands. Our data confirm a higher prevalence of NCD, including multimorbidity among senior workers. In particular, mental disorders and multimorbidity were associated with lower work ability, which in turn increases the risk of long-term sickness absence and premature exits from the labour market. As most Western societies require the workforce to prolong their working lives due to the mentioned demographic changes, it is therefore of vital importance to maintain and increase the working ability among senior workers, e.g., by preventing diseases and having a particular focus on workers with physically demanding work.

### 4.4. Strengths and Limitations

There are several strengths of our study. First, our primary outcome, i.e., work ability, was assessed by means of a validated single-item question originating from the WAI, which has been used in several studies measuring WAI [32,33]. Second, the generalizability of the results is strengthened by our use of model-assisted weights to ensure representativeness. Another strength of the study is the large sample of senior workers across both physically and mentally dominant job groups.

As this is a cross-sectional study, we cannot ascertain the causality of the observed associations, i.e., that high physical work demands and NCD lead to lower work ability. On the other hand, it is unlikely that lower work ability in itself leads to NCD and high physical work demands; however, having a known or diagnosed chronic disease may *per se* lead to perceived lower work ability. Questionnaire studies are generally prone to non-response bias if the responding participants differ from those who did not participate in this study, decreasing the generalizability to the general population. 

To account for potential non-response bias, we weighted the analyses using model-assisted weights based on national registers. The results of this study may be biased by the healthy worker effect [46], suggesting that those with severe chronic disease(s) may have already left the labour market. Thus, in the case of a healthy worker effect, our estimates may be considered conservative and may underestimate the associations.

NCDs were self-reported and could, therefore, be influenced by recall bias [47]. However, the questionnaire specifically stated that the participants should have received treatment or medication for their disease within the past year, which may reduce recall bias [31]. The validity of self-report questionnaires for measuring the prevalence of some diseases can be questioned. The question *Have you within the past year received treatment or medication for one or more of the following diseases?* may be difficult to answer if the diseases are similar, such as arthritis where it may be difficult to recognize the specific types of arthritis. Another observation for this study regarding recall bias is that the estimates for mental chronic disorders in this study might be conservative since the participants had to receive treatments or medication to meet one of the disorder criteria.

The prevalence of depression in this study was 3.7% while the prevalence of depression in the general population is around 3.3% [48], which indicates similarity regarding the prevalence. Depression often co-occurs with other disorders/diseases, such as heart disease and anxiety [49]. Having depression may, therefore, lead to multimorbidity rather than having only one disease.

## 5. Conclusions

In conclusion, this study among senior workers demonstrated that having one—irrespective of type—and particularly suffering from several NCDs was associated with reduced work ability and that high physical work demands influenced this association. Thus, senior workers with physically demanding jobs and several NCDs were at particularly increased risk of lowered work ability compared to sedentary workers with several NCDs. In addition, mental NCD appeared to influence work ability more when compared to somatic NCD. 

It is therefore important to focus on maintaining and increasing the work ability among senior workers by preventing diseases and adjusting physically demanding work tasks to the worker’s physical capacity in order to maintain a healthy and sustainable workforce for the long term. Furthermore, an increased focus on how companies benefit from having healthier workers who can maintain productivity and not leave the labour market prematurely is important knowledge for companies to prioritize health-promoting initiatives at the workplaces and, thereby, prolong the working life of the workers, which ultimately may contribute to maintaining a sustainable workforce in the future.

## Figures and Tables

**Table 1:** Demographic, lifestyle, diseases, and work-related characteristics of the included participants. Weighted means and percentages.

	*N*	Mean (95% CI)	SD	%
**Age (years)**	12,879	56.6 (56.5 to 56.7)	5.4	
**Sex**				
Male	7054			53.4%
Female	5825			46.7%
**BMI**	11,803	26.4 (26.3 to 26.5)	5.1	
**Highest completed education**				
Primary and/or high school	2573			20.7%
Short-or medium-term higher education	5421			41.5%
Long-term higher education	4781			37.8%
**Physical activity during leisure time**				
Mostly sedentary	1779			14.8%
Light exercise at least 4 h per week	7202			60.9%
Sports or heavy physical activity at least 4 h per week	2697			22.3%
Training and competing regularly and several times a week	233			2.0%
**Physical activity at work**				
Mostly sedentary work	5909			47.4%
Mostly physically demanding work	6264			52.6%
**Psychosocial work factors (0–100)**				
Influence at work	12,128	77.5 (77.1 to 77.9)	23.8	
Recognition from colleagues	12,111	77.0 (76.6 to 77.4)	22.5	
**Smoking**				
Yes, every day	1729			14.2%
Yes, but not every day	373			3.3%
Ex-smoker	4110			34.3%
No, never	5714			48.3%
**Number of NCD(s) within the last year**	11,913	1.2 (1.2 to 1.2)	1.6	
0	4710			40.6%
1	3451			28.7%
2	1965			16.4%
3	902			7.5%
≥4	885			6.9%
**Work ability (0–10)**	11,919	7.95 (7.92 to 7.98)	1.6	

BMI body mass index (kg/m^2^), *N* number, SE standard error, % percentage, and NCD(s) non-communicable disease(s).

**Table 2:** Least square mean differences (Mean diff.) and 95% confidence intervals (95% CI) for work ability in different NCD compared with no NCD. Pooled for all workers together.

Diseases	*N*	%	Mean Diff. (95% CI)
Burn-out syndrome	402	3.1	−1.46 (−1.61 to −1.32)
Stress	686	5.3	−1.18 (−1.29 to −1.07)
Anxiety	337	2.6	−1.15 (−1.31 to −0.99)
Chronic obstructive pulmonary disease (COPD)	203	1.6	−1.06 (−1.26 to −0.86)
Other long-term sickness/illness	738	6.0	−1.01 (−1.12 to −0.91)
Depression	484	3.7	−0.98 (−1.11 to −0.84)
Rheumatoid arthritis	618	5.0	−0.95 (−1.07 to −0.84)
Peptic ulcers	327	2.8	−0.93 (−1.08 to −0.78)
Osteoarthritis	1362	11.3	−0.86 (−0.94 to −0.79)
Apoplexy	183	1.5	−0.85 (−1.06 to −0.64)
Whiplash	104	0.9	−0.76 (−1.03 to −0.49)
Back diseases	1244	10.4	−0.72 (−0.80 to −0.64)
Ischemic heart disease	306	2.2	−0.69 (−0.86 to −0.52)
Cancer	318	2.5	−0.63 (−0.79 to −0.47)
Migraine/headache	916	8.1	−0.50 (−0.59 to −0.41)
Osteoporosis	236	1.9	−0.49 (−0.67 to −0.30)
Hearing impairment	731	5.7	−0.37 (−0.48 to −0.26)
Diabetes all types	622	4.2	−0.37 (−0.49 to −0.25)
Eczema	856	7.1	−0.36 (−0.46 to −0.27)
Cataract	227	1.7	−0.35 (−0.55 to −0.16)
Hypertension	2836	23.1	−0.31 (−0.37 to −0.25)
Asthma	748	6.5	−0.26 (−0.36 to −0.15)

*N* numbers, % weighted percentage, *Mean diff.* least square mean difference in work ability between the reference group (no diseases) and the included disease.

**Table 3:** The least square means and 95% confidence intervals (95% CI), as well as differences of the least square means, for work ability and the number of chronic diseases (multi-morbidity) stratified by job type.

	*N*	%	Model 1 Mean (95% CI)	Model 1 between-Group Mean Diff. (95% CI)	Model 2 Mean (95% CI)	Model 2 between-Group Mean Diff. (95% CI)
0 diseases Sedentary work	2439	20.2	8.45 (8.39 to 8.50)		8.44 (8.37 to 8.51)	
0 diseases Physical work	2264	20.4	8.12 (8.06 to 8.17)	0.33 (0.26 to 0.41)	8.19 (8.12 to 8.26)	0.25 (0.18 to 0.32)
1 disease Sedentary work	1669	13.6	8.24 (8.18 to 8.31)		8.28 (8.20 to 8.36)	
1 disease Physical work	1774	15.1	7.91 (7.85 to 7.97)	0.33 (0.25 to 0.42)	7.99 (7.91 to 8.06)	0.30 (0.21 to 0.38)
2 diseases Sedentary work	931	7.6	7.92 (7.83 to 8.00)		7.99 (7.90 to 8.09)	
2 diseases Physical work	1029	8.8	7.49 (7.41 to 7.57)	0.43 (0.31 to 0.55)	7.60 (7.50 to 7.69)	0.40 (0.28 to 0.51)
3 diseases Sedentary work	408	3.3	7.42 (7.29 to 7.55)		7.55 (7.41 to 7.68)	
3 diseases Physical work	494	4.2	7.18 (7.06 to 7.29)	0.24 (0.07 to 0.42)	7.33 (7.21 to 7.46)	0.22 (0.05 to 0.38)
≥4 diseases Sedentary work	327	2.5	7.04 (6.89 to 7.19)		7.20 (7.04 to 7.35)	
≥4 diseases Physical work	557	4.3	6.37 (6.26 to 6.49)	0.67 (0.48 to 0.85)	6.59 (6.47 to 6.72)	0.60 (0.42 to 0.78)

*N* numbers, % weighted percentage. Model 1 was adjusted for sex, age, and physical demands at work. Model 2 was additionally adjusted for education level, physical exercise, smoking, BMI, and psychosocial work factors (influence and recognition). The between-group mean difference is the difference between sedentary and physically demanding work in each group of numbers of diseases

## Data Availability

The authors encourage collaboration and the use of the data by other researchers. Researchers interested in using the data for scientific purposes should contact the project leader Lars L. Andersen, lla@nfa.dk.
